# Possible-sarcopenic screening with disturbed plasma amino acid profile in the elderly

**DOI:** 10.1186/s12877-023-04137-0

**Published:** 2023-07-12

**Authors:** Yushuang Duan, Kuan Tao, Zilong Fang, Yifan Lu

**Affiliations:** 1 School of Sports Medicine and Rehabilitation, Beijing Sport University, Beijing, China; 2 College of Rehabilitation, Weifang Medicine University, Weifang, China; 3 School of Sports Engineering, Beijing Sport University, Beijing, China

**Keywords:** Possible sarcopenia, Metabolomics, Plasma amino acid, Physical function, The elderly

## Abstract

**Background:**

The mass and strength of skeletal muscle decline with age, leading to its progressive dysfunction. High-throughput metabolite profiling provides the opportunity to reveal metabolic mechanisms and the identification of biomarkers. However, the role of amino acid metabolism in possible sarcopenia remains unclear.

**Objectives:**

The aim of this study included exploring variations in plasma amino acid concentrations in elderly individuals who have possible sarcopenia and further attempting to characterize a distinctive plasma amino acid profile through targeted metabolomics.

**Methods:**

A cross-sectional, correlational research design was used for this study. Thirty possible-sarcopenic elderly participants were recruited (*n* = 30), as determined by the Asian Working Group for Sarcopenia (AWGS). Meanwhile, a reference group of non-sarcopenic (sex-, age-, and Appendicular Skeletal muscle Mass Index (ASMI)-matched non-sarcopenic controls, *n* = 36) individuals was included to compare the potential differences in metabolic fingerprint of the plasma amino acids associated with sarcopenia. Both groups were conducted the body composition analysis, physical function examination, and plasma amino acid-targeted metabolomics. The amino acids in plasma were measured using ultra-performance liquid chromatography-tandem mass spectrometry (UPLC-MS–MS). Also, orthogonal partial least-squares-discriminant analysis (OPLS-DA) was applied to characterize the plasma amino acid profile.

**Results:**

With respect to Handgrip Strength (HGS), the Five-Repetition Chair Stand Test (CS-5), the Six-Minute Walking Test (6MWT), the arm curl, the 30 s-Chair Stand Test (CST), the 2-Minute Step Test (2MST), the Timed Up-and-Go Test (TUGT), there was a decline in skeletal muscle function in the possible-sarcopenic group compared to the non-sarcopenic group. The mean plasma concentrations of arginine, asparagine, phenylalanine, serine, lysine, glutamine, and threonine were significantly lower in the possible sarcopenia group, whereas cirulline, proline, serine, and glutamic acid concentrations were higher. According to the multi-analysis, glutamine, serine, lysine, threonine, and proline were determined as the potential markers that indicated possible sarcopenia.

**Conclusions:**

The findings characterize significantly altered plasma amino acid metabolisms in the elderly with possible sarcopenia, which aids to screening people who are at a high risk of developing condition, and motivating to design new preventive and therapeutic approaches.

## Introduction

A progressive decline in muscle strength and an increase in muscle fatigability evolve to a generalized deterioration of physiological function, and increase the rate of disability and dependency [1]. Sarcopenia, the term for the aging-related loss of skeletal muscle, has serious physiological and clinical repercussions [2–5]. Recent researches have indicated that the age-related decline in muscle strength is greater than what would be expected by the decline in muscle mass alone, with consequent decrease in the strength to mass ratio, also known as biomechanical muscle quality [6, 7]. Although the age-related decline in muscle strength is associated with the loss in muscle size [8], longitudinal studies discovered a 1.5 to 5 times greater decline in muscle strength compared with muscle size [9, 10]. In addition, there was a stronger correlation between muscle strength and disability compared to that between muscle mass and strength [11]. Evidence suggests that physical performance is influenced by muscular strength, and this relationship is curvilinear in the elder individuals [12–14]. Interestingly, decreasing muscle strength rather than muscle mass alone is a far stronger predictor of functional limitation and poor health in elderly [15]. Further strategies for early detection of those suffering from, or at a risk of developing sarcopenia, are advised by the Asian Working Group for Sarcopenia (AWGS) 2019 to enable necessary interventions in contexts lacking advanced diagnostic equipment [16]. In particular, AWGS 2019 introduces “possible sarcopenia”, defined as poor muscle strength with or without reduced physical performance, which is recommended for use in primary healthcare and preventive services [16]. The etiology of skeletal muscle mass and function loss may include satellite cell senescence, death of motor neurons, decreased activity of neuromuscular junctions, hormonal state, pro-inflammatory cytokines, impaired mitochondrial function, abnormal myokine synthesis, and weight loss accompanied by decreased appetite [3–5]. The actual cause of the reduction in skeletal muscle ability when aging, despite extensive research in this area, is still a mystery.

All organ systems in the human body are connected by plasma free amino acids (PFAA), which are abundantly circulated and whose profiles have been shown to be influenced by metabolic variations in specific organ systems induced by certain diseases [17]. Participants with physical frailty and sarcopenia that are characterized by physical function impairment [18], in particular, show distinct patterns of circulating amino acids and derivatives [19]. Meanwhile, lower plasma concentrations of the branched-chain amino acids (BCAAs) leucine and isoleucine defined the sarcopenic elderly Norwegian community dwellers [20]. Similarly, higher concentration of proline and a decline in plasma essential amino acids (EAAs) were seen in the elder Japanese with sarcopenia [21] and severe frailty, compared with non-frail peers [22]. Recent research from the Baltimore Longitudinal Study of Aging found that distinct amino acid signatures were associated with muscle mass in elderly with functional limitations and poor muscle quality [23, 24]. High levels of BCAA are related with a higher fat-free mass [25] and better skeletal muscle in elderly, respectively. Abnormal PFAA profiles in age-related diseases [23, 26, 27] indicated that skeletal muscular decline may be accelerated by defective PFAA metabolism. Furthermore, leucine-rich EAA supplementation plus physical exercise can increase the amount and the strength of skeletal muscle in sarcopenic elderly [28]. Therefore, the existence of an amino acid signature in advanced-aged individuals with weak skeletal muscle raises the possibility of specific metabolic and pathogenetic alterations. However, the discovery of biochemical indicators in such alterations has still attracted little attention.

Although the pathophysiology of aging is complicated and multifaceted, the major role for muscle recession suggests that biomarkers should be utilized to unveil underlying mechanisms and identify targets for interventions. As a result, amino acid profiling may be served as a potent analytical approach to explore the potential function of protein-amino acid networks in possible sarcopenia, especially when coupled with multivariate statistical analysis. The objectives of the current study are to characterize the plasma amino acids print and develop a metabolite predictor from possible sarcopenia, thus to provide an analytical explanation of complicated metabolic processes.

## Experimental procedures

### Study design

The cross-sectional study was based on baseline data from RCT study of the National Key Research and Development Program of China (RCTs registered at the Chinese Clinical Trial Registry on 19/10/2022, ChiCTR2200064801) conducting in October, 2022. Volunteers between the ages of 81 and 90 years were recruited from the Hua-Du Aged-care Center (Shandong Province, China). Sixty-six senior adults were enrolled after being informed of detailed explanation of procedures, risks of the investigation, and providing written informed consent. According to the AWGS 2019 recommendation, the possible sarcopenia was defined as low muscle strength with or without reduced physical performance: low muscle strength diagnostic cutoffs were handgrip (< 28.0 *kg*) for men and (< 18.0 *kg*) for women. Hence, participants were divided into two groups with possible sarcopenia and non-sarcopenia.

### Inclusion criteria

Age ranging from eighty-one to nighty; Ability to communicate with others; Ability to perform physical function test; No disease that might impair exercise performance.

### Exclusion criteria

To prevent issues that hinder the progress of the study, each participant was screened under scrutiny. Any of the following conditions happen could exclude a participant from fulfilling the subsequent experiments: Uncontrolled hypertension; Acute musculoskeletal injuries, joint contractures or internal metal implants such as total joint arthroplasty; Cardiovascular, pulmonary disorders or serious sequelae that could prevent them from engaging in exercise; Neurological impairment.

### Ethical procedure

All experiments were approved by the Ethics Committees of Beijing Sport University for Sports Science Experimental (trial number 2020082H). The codes of the China of Health were complied, according to *Ethical review measures for biomedical research involving human beings of Health, Science and Education* 200,717 (license number). All methods were performed in accordance with the relevant guidelines and regulations. The procedures and risks of the investigation were thoroughly explained to participants and written informed consent was obtained prior to participation.

### Assessments and procedures for data collection

#### Demographic information

A questionnaire was designed to collect demographic information (gender, age, illnesses, present medical issues, and lifestyle choices) and estimate dietary consumption, which has a great impact on metabolite. Experimenters ask information about the intake frequency of eggs, red meat, poultry, freshwater fishes, seafood, soybean products, milk. Meanwhile, blood specimens were collected from the participants and muscle mass, physical function examination were also performed. Basic information, physical function indexes and plasma amino acids metabolites were compared between possible sarcopenic and non-sarcopenic control groups. The data used in this study were anonymized and masked for analysis.

#### Body composition and physical function measurements

InBody S10 (InBody Co. Ltd, Seoul, Korea) was used to estimate muscle mass *kg*/m^2^ in the standing position. After height and weight were measured, four electrodes were attached to both upper and lower extremities to obtain appendicular and trunk muscle mass. ASMI was calculated by dividing the upper and lower extremities the muscle mass by height squared in meters [29]. According to the literature, a wide range of physical performance tests are used to test physical function, including Handgrip Strength (HGS) [30], Five-Repetition Chair Stand Test (CS-5) [31], 6-Minute Walking Test (6MWT) [32], arm curl [33], 30 s-Chair Stand Test (CST) [33], 2-Minute Step Test (2MST) [33] and the 8-Feet Timed Up-and-Go Test (TUGT) [33]. Before experiments, the investigator informed participants of the procedure and performed a demonstration. In order to avoid fatigue build-up, the upper- and lower-body tests were performed by turns and the interval between each test was approximately 3 min.

During HGS (using a dynamometer, CAMRY EH101) assessment session, participants were asked to stand upright with their feet hip-width apart and to face forward with hands holding a dynamometer in a neutral position (elbows not extended and the index finger flexed with 90°) until the elbows were fully extended. All participants were required to grip at their maximum force for 3 s, and 60 s between each set. In CS-5 test session, participants were asked to fold their arms across their chest (armrests were not used) and stand up from the standardized armless chair (0.47 m in height) during pre-experiment test, and performed chair stands for 5 times in a timely manner. 6MWT test session was performed as participants walked at their maximum speed along a 50-m-long rectangular field, which was ceased if participants were exhaustive. TUGT session aimed at assessing dynamic balance ability. Participants were seated with hands on thighs and feet flat on the floor. They were instructed to walk as quickly as possible back-and-forth towards a cone that was placed 3 m away, and recover to a fully seated position on the chair. CST and arm curl sessions covered tests on lower-body strength. Participants should complete stand-and-sit challenge (CST) and weight-lifting trials (arm curl session, 8-lbs dumbbell for male and 5-lbs dumbbell for female) as many times in 30 s. The final session, 2MST, is an alternative aerobic endurance test. Participants should raise each knee to a point midway between the patella (kneecap) and iliac crest (top hip bone) in 2 min.

#### Blood sample collection and plasma amino acids quantification

For measures of plasma amino acids metabolites, blood samples were collected from the forearm vein into vacutainers containing lithium-heparin after an overnight fast at the same day with physical function examination. The plasma-fraction was then obtained by centrifugation at 3,000 $rpm$ for 30 min at 4 ℃. The samples were then immediately stored at -80 ℃ for further analysis [34].

The amino acids in plasma were measured using ultra-performance liquid chromatography-tandem mass spectrometry (UPLC-MS–MS). Samples were analyzed on a QTRAP 5500 LC–MS/MS system (SCIEX, Framingham, MA) equipped with a Waters UPLC (Waters, USA) [35].

For the quantification of amino acids, 40 *μL* plasma was mixed with 20 μL stable-isotope-labeled internal standard (IS) in sulfosalicylic acid to precipitate proteins, and this process was then vortexed and centrifuged (4000 $rpm$, 4℃, 20 min). Chromatographic separation was achieved on an HSS T3 column (100 × 2.1 mm, 1.8* μm*, Waters, USA), and the column temperature was maintained at 40 $^\circ{\rm C}$. The UPLC system employed a gradient elution program consisting of water with 0.1% formic acid (mobile phase A) and acetonitrile (mobile phase B). The linear gradient was optimized and was as follows: 0.3–6 min, 2% to 40% B; 6–9 min, 40% B, 9–9.5 min for 40% to 90% B, with a 0.5 $ml/min$ flow rate. Calibration was achieved by spiking plasma with various concentrations of amino acid standards. Data were further analyzed using MultiQuant software (SCIEX, USA) [35].

## Statistical methods

### Descriptive statistics

Mann–Whitney U-test was implemented to evaluate the demographic information, food frequency, physical function and plasma amino acid concentration and Chi-square test to alcohol-drinking history, disease history and exercise habit between the possible-sarcopenic and non-sarcopenic group with IBM SPSS software 23.0. Data are presented as mean $\pm$ standard deviation (M $\pm$ SD), with statistical significance value $p<0.05$.

### The plasma amino acid analysis

The data generated by the plasma amino acid metabolomics were then imported separately into the SIMCA-P + 14.1 software package. We used principle component analysis (PCA) and orthogonal partial least-squares-discriminant analysis (OPLS-DA) to identify differences of plasma amino acids. The contribution of each metabolite ion to the discrimination of each group is reflected in the variable importance in projection ($vip$) obtained during OPLS-DA processing. Variables ($vip>1$ and $p<0.05$), along with the metabolites with statistical significance through multivariate and univariate analyses ($vip>1$ and $p<0.05$) were sufficient to distinguish the possible sarcopenia group from the control one.

## Results

### The characteristics of participants information and physical function

Information such as ages, food frequency, alcohol-drinking history, disease history, exercise habit from questionnaire and physical function characteristics for the entailed participants between possible sarcopenia and non-sarcopenia were listed in Tables 1 and 2. There was no discernable difference in ages, food frequency, alcohol-drinking history, disease history, and Appendicular Skeletal muscle Mass Index (ASMI) between two groups. However, physical function and exercise habit was poorer in participants who have possible sarcopenia than in controls, as expected ($p<0.01$).

**Table 1:** The main characteristics between two groups

Basic information	Possible sarcopenia (*n* = 30)	Non-sarcopenia (*n* = 36)	${\varvec{p}}$-value
age (y)	85.77 ± 3.53	84.69 ± 2.73	0.169
coarse cereals	20.00 ± 4.75	19.67 ± 4.60	0.78
eggs	17.66 ± 6.19	16.67 ± 2.24	0.238
red meat	20.24 ± 17.57	21.22 ± 4.44	0.476
poultry	20.59 ± 18.17	21.28 ± 5.37	0.063
freshwater fishes	23.17 ± 8.51	24.56 ± 4.77	0.741
seafood	25.38 ± 8.33	25.61 ± 3.24	0.151
soybean products	23.59 ± 7.06	24.22 ± 4.16	0.811
milk	18.34 ± 7.07	17.61 ± 4.63	0.824
alcohol-drinking history (yes or no)	2/25	4/30	0.570
hypertension (yes or no)	15/12	21/13	0.624
hyperlipidemia (yes or no)	3/24	5/29	0.68
diabetes (yes or no)	6/21	6/28	0.655
cardiopathy (yes or no)	8/19	12/22	0.640
exercise habit (yes or no)	10/17	27/7	0.001

The results from food frequency questionnaire (collected weekly) were listed, and variables were analyzed by Mann–Whitney U tests and expressed as *M* ± *SD* (mean $\pm$ standard deviation), which showed significant difference between two groups (*p* < 0.05). Alcohol-drinking history, disease history and exercise habit were represented by example (*n*) and analyzed by Chi-square test, significantly different between two groups (*p* < 0.05)

**Table 2:** The physical function values between two groups

Physical performance	Possible sarcopenia (*n* = 30)	Non-sarcopenia (*n* = 36)	*p*-value
ASMI	6.13 ± 0.97	6.19 ± 1.42	0.835
HGS	15.60 ± 5.24	25.21 ± 6.95	0.00
CS-5	15.94 ± 2.24	9.16 ± 1.70	0.00
6MWT	278.15 ± 78.52	416.36 ± 99.51	0.00
Arm curl	13.03 ± 4.75	17.86 ± 7.29	0.003
CST	8.20 ± 2.72	16.19 ± 2.65	0.00
2-MST	45.50 ± 17.29	89.08 ± 16.27	0.00
TUGT	17.85 ± 6.34	9.78 ± 3.14	0.00

Data were represented as *M* ± *SD* (mean $\pm$ standard deviation). Variables were analyzed by Mann–Whitney U tests, significantly different between two groups (*p* < 0.05). ASMI (Appendicular Skeletal muscle Mass Index), HGS (handgrip strength, *kg*), CS-5 (Five-Repetition Chair Stand Test, *s*), 6MWT (the six-minute walking test, *m*), Arm Curl (repetitions), CST (the 30 s-chair stand test, repetitions), 2MST, (2-min step test repetitions), TUGT (timing up-and-go test, seconds). Gestational age of participants stands for the time at which plasma samples were collected

### Plasma amino acids concentration

Nine plasma amino acids showed the substantial variations between the two groups. The general pattern of citrulline, glutamic acid, and proline plasma concentrations generally increased in possible-sarcopenic group. The concentrations of seven (out of twenty amino acids) decreased with possible sarcopenia, namely arginine, serine, asparagine, glutamine, threonine, phenylalanine and lysine (Table 3).

**Table 3:** Concentration of Plasma amino acids between two groups

Plasma amino acid	Possible sarcopenia (*n* = 30)	Non-sarcopenia (*n* = 36)	*p*-value
Alanine	430.24 ± 144.98	422.26 ± 186.74	0.849
Arginine	68.5 ± 31.34	83.43 ± 27.60	0.044
Asparagine	29.97 ± 8.74	39.16 ± 8.75	0.000
Aspartic acid	7.03 ± 5.87	5.66 ± 4.47	0.285
Citrulline	45.61 ± 17.28	37.02 ± 16.09	0.041
Glutamine	329.39 ± 81.31	397.80 ± 113.72	0.008
Glutamic acid	81.51 ± 41.75	63.57 ± 34.31	0.001
Histidine	59.90 ± 18.78	54.70 ± 12.31	0.199
Isoleucine	74.72 ± 26.59	75.40 ± 24.73	0.914
Leucine	126.54 ± 43.96	119.95 ± 34.45	0.507
Lysine	204.04 ± 67.04	228.28 ± 56.25	0.022
Methionine	24.05 ± 8.12	25.09 ± 5.94	0.551
Ornithine	53.85 ± 25.33	54.38 ± 24.33	0.931
Phenylalanine	61.93 ± 20.52	69.21 ± 14.20	0.012
Proline	358.79 ± 244.53	228.22 ± 111.63	0.010
Serine	67.90 ± 30.67	86.63 ± 37.80	0.030
Threonine	99.77 ± 30.24	119.08 ± 37.38	0.026
Tryptophan	45.67 ± 12.05	41.68 ± 10.60	0.157
Tyrosine	70.82 ± 21.10	69.11 ± 17.50	0.719
Valine	215.83 ± 67.12	222.37 ± 58.40	0.673

Data were expressed as *M* ± *SD* (mean ± standard deviation). Variables were analyzed by Mann–Whitney U tests, significantly different between two groups *p *<0.05

### Classification of plasma amino acid based on OPLS-DA

**Fig. 1:**
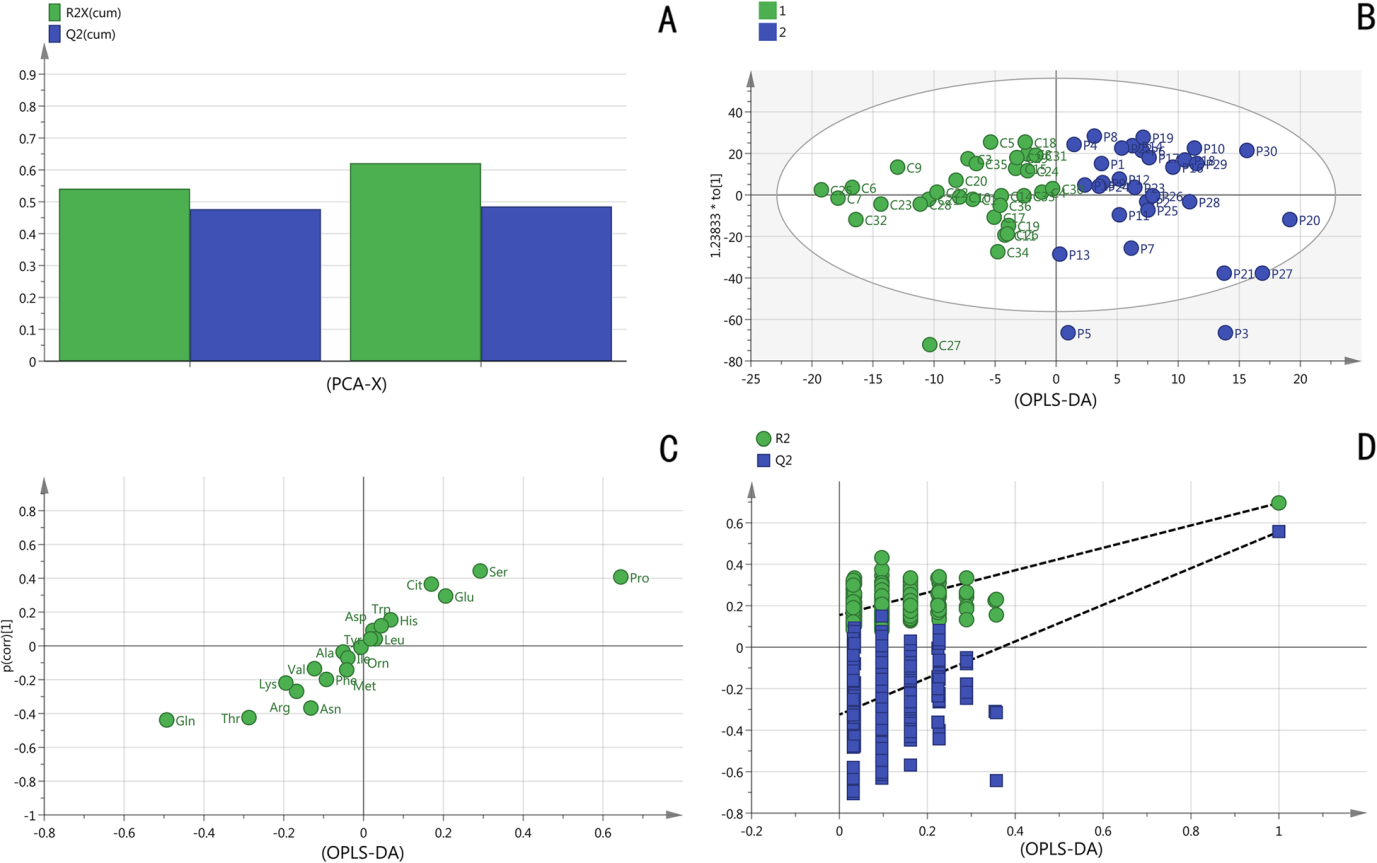
**A** PCA (principal component analysis) Scores plot between two groups. **B** Scores plot showing the separation of participants according to the plasma concentrations of amino acids and derivatives in the space spanned by the two latent variables (R2X and R2Y), as determined by orthogonal partial least-squares-discriminant analysis (OPLS-DA). 1 = non-sarcopenia, 2 = possible sarcopenia.** C** Loadings plot. **D** Permutation testing

In order to verify the existence of distinct patterns of amino acids in participants with possible sarcopenia, a OPLS-DA classification model was constructed and validated. Age, gender and ASMI did not significantly differ between the two groups in this study. Besides, diversity in exercise, nutrition, and living conditions have effects on metabolomics results. In order to reduce the impact of interference factors on the experimental results, OPLS-DA, which combines partial least squares discriminant analysis and orthogonal signal correction, can screen the differential variables by removing individual differences. The PCA score plot (Fig. 1A) which used two components (R2Xcum = 0.621, Q2cum = 0.485), exhibited the separate trends comparing maternal plasma from the two groups. These results indicate that plasma contains a particular pattern of amino acids. The twenty amino acids in plasma samples were subjected to OPLS-DA analysis, with the model parameters being R2Xcum = 0.787, R2Ycum = 0.727, Q2cum = 0.582. OPLS-DA model shows great explanatory and predictive ability, as witnessed by considerable differences of plasma amino acid profile in possible sarcopenia, which were completely distinguishable from the score (Fig. 1B and C). In addition, permutation testing proves that the model is stable and reliable without over-fitting (Fig. 1D).

Further, to identify the metabolites that were mostly responsible for discriminating between two groups, the values of $VIP$ indices were examined. The variables with $VIP>1$ were listed in Table 4. The amino acid-targeted metabolomic profiling identified five amino acids altered in possible sarcopenia, of which four were markedly down-regulated, including glutamine, threonine, serine, lysine and threonine, whereas proline were noticeably up-regulated.

**Table 4:** Plasma amino acids signatures

Metabolites	*vip*-value	Possible sarcopenia	Non-sarcopenia	*p*-value
Glutamine	1.81	329.39 ± 81.31	397.80 ± 113.72	0.008
Proline	2.68	358.79 ± 244.53	228.22 ± 111.63	0.010
Serine	1.11	67.90 ± 30.67	86.63 ± 37.80	0.030
Lysine	1.03	204.04 ± 67.04	228.28 ± 56.25	0.022
Threonine	1.01	99.77 ± 30.24	119.08 ± 37.38	0.026

Plasma concentrations of discriminant analytes, variable importance in projection ($vip$) values between possible sarcopenia and non-sarcopenia. Plasma concentrations were shown as *M* ± *SD *(mean $\pm$ standard deviation)

## Discussion

Impaired mobility and functional decline are two of the most prominent physiological changes associated with aging [36]. While there are many factors that contribute to aged-related physical limits, one of the most noticeable factors is that skeletal muscle performance progressive declines and deteriorates over time [37]. Skeletal muscle strength, power, endurance, and contractile function gradually reduce with aging [11, 38–41]. Similar to this, we have also observed that skeletal muscle function gradually declines as the likelihood of sarcopenia increases through the tests for HGS, CS-5, 6MWT, arm curls, CST, 2MST, TUGT.

Age-related acceleration of these declines in skeletal muscle physiological function onsets between the ages of 30 and 40 years [42]. Protein metabolism throughout aging is responsible for a number of physiological and functional abnormalities in skeletal muscle, which are aggravated by decreased dietary protein intake and delayed protein synthesis responses to stimuli. Additionally, amino acids serve as both nutrients and regulators for the production of various macromolecules, including proteins, which are essential to maintain both physical and mental functions. There have been reports on the relationships between circulatory amino acid concentrations and muscle mass and strength [25, 43, 44], which are consistent with studies demonstrating that BCAAs are markers of muscle mass (or strength) in healthy individuals [43, 44]. Our findings revealed that the pattern of plasma amino acid concentrations had changed, and that possible sarcopenia were associated with the presence of distinctive metabolites. Plasma levels of free amino acids are in a dynamic equilibrium that is altered by the daily protein intake [45]. The variations in plasma amino acid profile would be attributed to possible sarcopenia according to the food frequency questionnaire. To put it another way, systemic amino acid metabolism is different in elderly compared to elder persons with reasonably normal skeletal muscle strength and function. This is the first study that, to our knowledge, has linked possible sarcopenia with the plasma amino acid metabolomics profiles.

Studies have shown that perturbations of amino acid metabolism are widespread in elderly with skeletal muscle hypofunction [46]. In line with these observations, we discovered that possible sarcopenia was associated with lower circulating levels of the EAA including serine, lysine and threonine. In addition, there was a decreasing trend of valine for possible sarcopenia, yet this difference was not statistically significant. Both sexes were found to have lower serum levels of a number of EAAs as they aged [22]. Thus, amino acid metabolic disorders may also be risk factors for the development of possible sarcopenia. When there is a lack of amino acids, skeletal muscle proteins are degraded together with amino acids, which incurs severe problems of skeletal muscle health degradation. According to reports, EAA is primarily responsible for the anabolic effect of amino acids on muscle proteins [47]. By activating the mammalian target of rapamycin complex, EAAs, particularly BCAAs, influence protein synthesis and nutritional status [48, 49]. Meanwhile, we found significantly decreased glutamine levels and markedly increased glutamic acid levels in possible sarcopenia. Glutamine has been shown to regulate mTORC1 by affecting autophagy, transamination and other specific functions, which is considered an essential tissue size and mass regulator, either in healthy or ill patients [50, 51]. The mTORC1 pathway is highlighted as a key therapeutic target to prevent sarcopenia by the age-related alterations in skeletal muscle [52–55]. In fact, in skeletal muscle, mTORC1 activation regulates protein synthesis and regulates skeletal muscle mass [56]. Moreover, the activity of amino acids, particularly leucine, as anabolic inducers in muscle cells is hampered by mTOR when glutamine is not available [51]. Another reason for the formation and progression of sarcopenia is protein anabolic resistance brought on by insulin resistance [57, 58]. Cheng et al. reported that plasma glutamine, glutamic acid, and the glutamine (glutamic) acid ratio were highly correlated with insulin resistance traits in two different cohorts, the Framingham Heart Study and the Malmö Diet and Cancer Study [59]. These findings suggest the possibility that skeletal muscle mass and function are affected by the decreased glutamine concentrations in the elderly. In order to delay the deterioration of possible sarcopenia with aging, glutamine depletion may have a role in both prevention and treatment. Besides, our multivariable analysis revealed that a higher plasma concentration of proline was the variable associated with possible sarcopenia. The results corroborate those that were found in earlier research on sarcopenia in the elderly [21]. Ilaiwy et al. also observed that muscle cell atrophy was related to the elevated proline concentrations in culture media in vitro, again agreeing with our findings [60]. Likewise, a high concentration of proline has also been confirmed to induce oxidative damage to protein, lipids, and DNA in rats [61].

In summary, a low-quality protein diet or malabsorption may have reduced the amount of EAAs, which limits protein synthesis and may have therapeutic effects on the quality of skeletal muscle in the elderly with possible sarcopenia. A study on severely fragile elderly patients found that low plasma levels of EAAs characterized the amino acid profile, and were caused by poor nutritional status [22]. Furthermore, leucine-rich EAA supplementation combined with physical exercise can increase the amount and strength of skeletal muscle in sarcopenic groups [28]. In light of this, our findings are valuable for interventions therapies of possible sarcopenia, particularly EAA supplementations to prevent the development of skeletal muscle recession. The profile appears to indicate metabolic abnormalities that are subsequent to potential sarcopenia, which is an another more-likely explanation. The anaplerotic pathway of the tricarboxylic acid cycle receives amino acids as a source of energy for metabolism. When the elderly with possible sarcopenia are malnourished, amino acids, especially proline, are produced by proteolysis in skeletal muscle and used for energy metabolism. In contrast, they are not metabolized via this pathway in healthy elder individuals with sufficient carbohydrate intake.

At the same time, when considering physical dysfunction as the ultimate outcome of muscle failure [62], numerous cellular and molecular changes may occur. Several factors have been pinpointed [19, 63, 64] to explore the pathways involved in possible sarcopenia pathophysiology, yet the underlying mechanisms remain unclear. Physical performances shows great heterogeneity for individuals from various socioeconomic backgrounds [65–67], implying that age- or country-specific cut-off values might be incorporated to identify physical dysfunction [68]. It is assumed that biological pathways leading to pathologic conditions causes altered concentrations of specific amino acids, which aids to elucidate mechanistic paths for the skeletal muscle degradation. Muscle strength decline, rather than muscle mass alone, is a fairly convincing predictor of functional limitation and poor health in the elderly [15]. Motivated through the results in this study, we aim to reveal the risks of early identifications for developing sarcopenia, developing preventive strategies and novel treatments.

Skeletal muscle changes are prevalent in the elder people as an age-related process, influenced not only by contemporaneous risk factors, but also by hereditary and lifestyle factors. It has been shown that exercise habits are significant predictors of the appendicular muscle mass and skeletal muscle mass index [69–71]. Our results also indicate that engaging in daily physical activity and sports may play an important role in maintaining appropriate levels of strength. Further researches are required to clarify the relationship between skeletal muscle quality, lifestyle factors and circulating amino acids in the context of possible sarcopenia. Also, studies on intervention approaches are needed to optimize lifestyle habits for skeletal muscle recession.

There were some limitations to this study. First, bias on participants’ selection cannot be ruled out. Given that the participants were volunteered from two regional healthcare center and not chosen at random, some findings in this study are inevitably biased. Second, since this was a cross-sectional study, we were unable to relate our findings to the likelihood of possible sarcopenia in the elderly. Finally, dietary variations among participants (since no nutrition restrictive requirements for this study), particularly differences in protein intake, may partially affect the variability to concentrations of amino acids.

## Data Availability

The dataset supporting the conclusions of this article is included within the article. All data generated and analyzed during this study are included in this published article.
